# Identification of the hASCT2-binding domain of the Env ERVWE1/syncytin-1 fusogenic glycoprotein

**DOI:** 10.1186/1742-4690-3-41

**Published:** 2006-07-04

**Authors:** Valérie Cheynet, Guy Oriol, François Mallet

**Affiliations:** 1 UMR 2714 CNRS-bioMérieux, IFR128 BioSciences Lyon-Gerland, Ecole Normale Supérieure de Lyon, 69364 Lyon Cedex 07, France

## Abstract

The cellular HERV-W envelope/syncytin-1 protein, encoded by the envelope gene of the ERVWE1 proviral locus is a fusogenic glycoprotein probably involved in the formation of the placental syncytiotrophoblast layer. Syncytin-1-induced *in vitro *cell-cell fusion is dependent on the interaction with hASCT2. As no receptor binding domain has been clearly defined in the SU of neither the HERV-W Env nor the retroviruses of the same interference group, we designed an *in vitro *binding assay to evaluate the interaction of the HERV-W envelope with the hASCT2 receptor. Using truncated HERV-W SU subunits, a region consisting of the N-terminal 124 amino acids of the mature SU glycoprotein was determined as the minimal receptor-binding domain. This domain contains several sub-domains which are poorly conserved among retroviruses of this interference group but a region of 18 residus containing the SDGGGX_2_DX_2_R conserved motif was proved to be essential for syncytin-1-hASCT2 interaction.

## Findings

The cellular HERV-W envelope protein, also named syncytin-1, is a fusogenic glycoprotein probably involved in the formation of the placental syncytiotrophoblast layer [1-3]. This protein, encoded by the envelope gene of the ERVWE1 proviral locus [4] is synthesized as a gPr73 precursor and is specifically cleaved into two mature proteins, a gp50 surface subunit (SU) and a gp24 transmembrane subunit (TM) [5]. The HERV-W Env glycoprotein is phylogenetically related to an interference group of retroviruses [6] that includes feline endogenous virus (RD114), baboon endogenous virus (BaEV), simian retroviruses (SRV-1, SRV-2), avian reticuloendotheliosis viruses (REV-A) and spleen necrosis virus (SNV) [7]. All retroviruses of this group share a common cell surface receptor, the human sodium-dependent neutral amino acid transporter type 2 (hASCT2) [8,9]. Accordingly, syncytin-1-induced *in vitro *cell-cell fusion is dependent upon interaction with hASCT2 [1] and also with the amino acid transporter hASCT1 [10]. Moreover, HERV-W Env confers host cell resistance to infection by SNV [11]. To date, no RBD has been clearly defined in the SU of the HERV-W Env interference group. Within the type C mammalian retroviruses, the SU subunit harbors three contiguous regions consisting of the SU amino-terminal receptor binding globular domain [12-14], a proline-rich region (PRR) and the TM-interacting SU carboxy-terminal domain. A similar RBD has been also delineated in the SU N-terminal region of HTLV1, HTLV2 [15,16] and predicted in Mammary Tumor Virus [17] and Bovine Leukemia virus envelopes [18].

In this study, we have designed an *in vitro *binding assay to evaluate the interaction of HERV-W envelope-derived soluble SU domains with the hASCT2 receptor. We have identified a 124 residue region of the mature SU as the minimal domain that interacts with the hASCT2 receptor.

**Figure 1:**
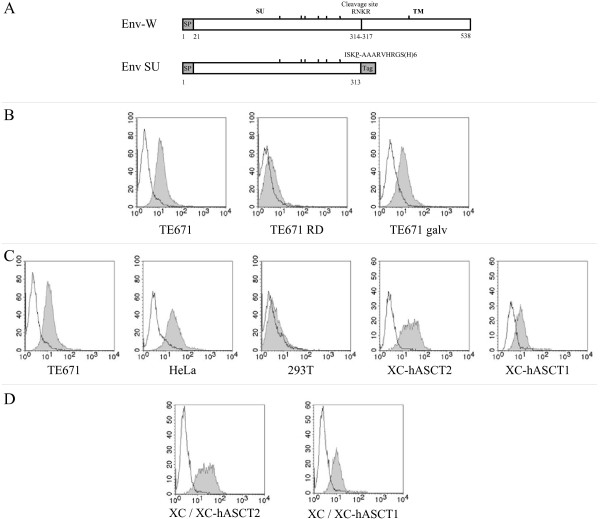
**Cell surface binding assays of soluble SU**. A) Schematic representation of HERV-W envelope protein (Env-W) and SU protein (EnvSU). Surface (SU, 1–313) and transmembrane (TM, 318–538) domains and the consensus furin cleavage site (RNKR, 314–317) are indicated. (^|^) N-glycosylation sites) [5]. Gray boxes indicate the signal peptide (SP) and a 15-amino-acid AAARVHRGS-H6 sequence (Tag). Residues are numbered starting from the initiation methionine. The ISKP sequence corresponds to the carboxy-terminal amino acid residues of the native SU included within the construct, and the P underlined amino acid corresponds to numbered residue located just upstream from the tag. B) Interaction of the soluble SU with the type D mammalian receptor. Soluble SU protein was secreted from HEK293T cells transfected with the SU domain expression vector and cultured for 24 h in medium without serum. Parental TE671, TE671 RD (hASCT2-blocked cells) and TE671 GALV (PiT1-blocked cells) cells were incubated at 37°C for 1 h in supernatants with (shaded) or without (white) soluble SU protein, collected from transfected or native HEK293T cells, respectively. Binding of the tagged soluble SU onto the cells was detected by incubating 1 h at 4°C the cell-protein mixture with an anti-histidine antibody (anti-RGS(H4)-Mab; Qiagen) in PBA (PBS with 2% fetal calf serum and 0.1% sodium azide). Cells were washed once and incubated with fluorescein isothiocyanate-conjugated antibody (DAKO) for 1 h at 4°C in PBA. The viable cells were analyzed by flow cytometry. C) Binding assay of SU soluble protein with target cells expressing various levels of hASCT1 and hASCT2 receptors. TE671, HeLa, HEK293T, XC-hASCT2 and XC-hASCT1 cells were incubated at 37°C for 1 h in HEK293T cell culture supernatants with (shaded) or without (white) soluble SU protein as indicated above. Binding assays were performed as described in 1B. D) Binding of SU soluble protein is restricted to human ASCT receptors. Soluble SU protein were incubated at 37°C for 1 h with parental XC rat cells (white) and XC-hASCT2 or XC-hASCT1 (shaded) stable cells Binding assays were performed as described in 1B.

The HERV-W soluble SU subunit was constructed, expressed and tested as follows. Firstly, we used the phCMV-EnvW (clonePH74) expression vector containing the complete *env *gene as a template to generate soluble gp50 protein. In order to produce a soluble tagged gp50 protein, a full-length domain (phCMV-EnvSU) was constructed as a fusion protein containing a C-terminal VHRGS(H6) sequence located just downstream from the RNKR cleavage site replaced with an AAAR sequence (Figure 1A). Secondly, Env protein was recovered from cell culture medium without serum after transient expression in HEK293T cells. Finally, the binding assay using the soluble SU subunit was performed as previously described by Lavillette [19]. Briefly, target cells expressing the relevant receptor(s) were incubated with the supernatant containing the soluble envelope. The cells were stained with anti-histidine antibody (anti-RGS(H4) Mab; Qiagen) to detect soluble SU and analyzed by a fluorescence-activated cell sorter (FACS Calibur, Becton Dickinson).

In order to verify that the soluble SU protein behaves similarly to the SU subunit within the context of the native syncytin-1, we first examined the interaction of SU protein with hASCT2 receptor using parental and receptor-blocked TE671 human cells as target cells (Figure 1B). Indeed, TE671 cells were originally used to demonstrate the fusogenic properties of the native syncytin-1 protein. The target cells were TE671 subclones stably expressing the envelope glycoproteins derived from the GALV and RD114 retroviruses that respectively recognize the PiT-1 [20] and hASCT2 (also named RDR for type D mammalian retrovirus receptor) [8,9] receptors, thus blocking the accessibility of exogenously presented retroviral envelopes to these receptors, as previously described [1]. As expected, the soluble SU protein bound to TE671 parental cells, which express the receptors for several retrovirus groups, to PiT1-blocked cells, but not to hASCT2-blocked cells. These data confirmed that soluble SU protein, similarly to syncytin-1, recognizes the type D mammalian retrovirus receptor expressed on human cells and thus behaves like retroviral glycoproteins of the interference group.

To infer the sensitivity and specificity of the cell surface binding assay, soluble SU subunit was bound to a panel of target cell types expressing different combinations of hASCT1 and hASCT2 receptors: TE671 cells which contain hASCT1 and hASCT2 [10], HeLa cells which contain hASCT2 but not hASCT1 [21], HEK293T cells which contain small amounts of both hASCT1 and hASCT2 [22], and XC rat cell clones stably expressing either hASCT2 or hASCT1 human receptors and derived from XC cells by transfection of the pCD3.1VHR16/14 [9] and pCDNA3.1-hASCT1 [10] expression plasmids, respectively (Figure 1C). In this assay, binding to HeLa cells was slightly higher than to TE671 cells, demonstrating the different expression and/or distribution of the hASCT2 receptor on these target cells. In contrast, the HEK293T cells weakly bound SU protein. Using XC-hASCT1 and XC-hASCT2 cells (Figure 1C), we showed that the SU subunit could efficiently bind both to hASCT1 and hASCT2 receptors, as observed for both cell-cell fusion and infection assays [10].

Finally to exclude any potential binding of soluble SU onto non-human ASCT-related receptor expressed by parental XC cells, we compare the binding efficiency of SU between parental XC cells and XC-hASCT2 or XC-hASCT1 cells (Figure 1D). As expected, the SU protein bound to XChASCT2 and XChASCT1 cells but not XC parental cells [1].

**Figure 2:**
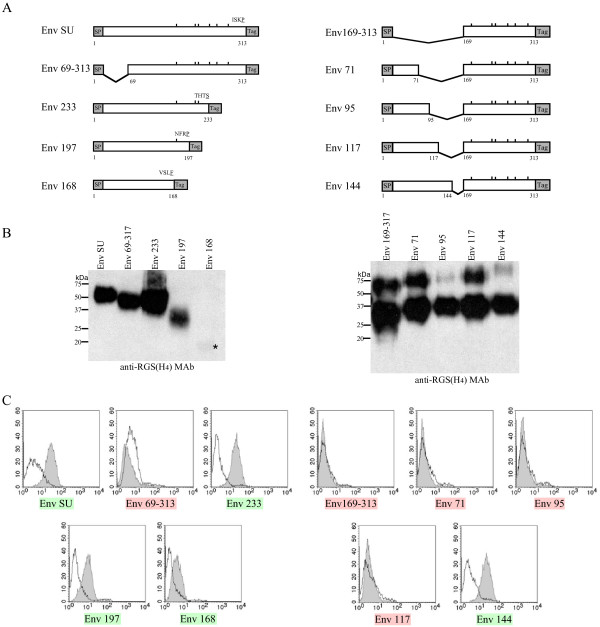
**Cell surface binding assays of SU and truncated proteins**. A) Schematic representation of SU and truncated proteins. Gray boxes indicate signal peptide (SP) and 15-amino-acid AAARVHRGS-H6 sequence (Tag). Residues are numbered starting from the initiation methionine. ISKP, THTS, NFRP and VSLF sequences correspond to the carboxy-terminal amino acid residues of the native SU included within each construct, SU, Env233, Env197 and Env168, respectively. The underlined amino acids correspond to the numbered residue located just upstream from the tag. B) Detection of SU and truncated proteins in culture medium. Culture supernatants were collected from HEK293T cells transfected with either the SU domain or truncated envelope expression vectors as described in fig 1B. 20 μl of supernatant was denatured (0.5% sodium dodecyl sulfate [SDS], 1% β-mercaptoethanol) at 100°C for 10 min and analyzed by SDS-10% polyacrylamide gel electrophoresis. Blots were probed with with an anti-histidine antibody (anti-RGS(H4)-Mab; Qiagen). Blots were developed using horseradish peroxidase-conjugated antibodies (Jackson) together with an enhanced chemiluminescence kit (Amersham Pharmacia). * indicates the position of Env168 SU protein. C) Identification of the receptor binding domain. SU and truncated soluble proteins were incubated at 37°C for 1 h with XC-hASCT2 cells (shaded) or with parental XC cells (white). Binding assays were performed as described above. The binding capacity of each recombinant protein onto hASCT2 receptor is depicted with a green (efficient) or red (inefficient) highlighted name.

As the features of the SU recombinant protein have been shown to be similar to the native syncytin-1 protein, we designed a series of N- and C-terminal truncation mutants in order to identify the RBD of syncytin-1 (Figure 2A). SU versus truncated SU-derived proteins were tested for their ability to bind hASCT2 receptor-presenting cells (XC-hASCT2) by flow cytometry and XC parental cells were used as a control. Domains of the SU subunit were generated by PCR, subcloned into the phCMV-EnvSU expression vector and sequenced. EnvSU, Env69–313, Env233, Env197 and Env168 proteins were expressed in transfected HEK293T cells and the expression level evaluated by Western blot analysis (Figure 2B). EnvSU, Env233, Env197 were expressed, secreted into the cell culture medium and exhibited similar binding efficiencies (Figure 2C), demonstrating that the N-terminal 176 residues of the mature SU are sufficient for hASCT2 recognition. Interestingly, deletion of the 22–68 region leads to the loss of receptor recognition, also suggesting its implication in the RBD. The Env168 mutant was poorly detected in the supernatant, suggesting problems of expression, secretion and/or stability for this truncated protein. Nevertheless, it still showed a weak binding capacity. Thus, in order to obtain similar quantities of soluble recombinant proteins derived from smaller N-terminal regions, we decided to fuse those N-terminal fragments with a carboxy-terminal domain (residues 169 to 313) of the SU protein (Figure 2A) which is unable to bind receptor (see below). A homogenous expression level of Env169–313, Env71, Env95, Env117 and Env144 truncated envelopes in cell culture supernatants was confirmed by Western blot analysis (Figure 2B). Only the Env144 protein bound to its receptor (Figure 2C). In order to verify if the receptor binding capacity of Env144 requires an interaction between the amino- and carboxy-terminal domains of this SU-derived construct, we substituted the 169–313 amino-acids in Env144 protein by an heterologous sequence from EGF coding for 53 amino-acids. The binding of the fusion protein (Env144-EGF) was similar to that observed for Env144, demonstrating that the carboxy-terminal part of the SU subunit does not contribute to receptor binding (data not shown).

**Figure 3:**
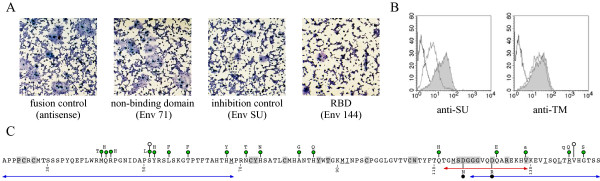
**Validation of RBD functionality**. A) Cell-cell fusion inhibition by the RBD. The wild-type HERV-W plasmid was transfected in TELCeB6 wild-type envelope producer cells phenotypically characterized by a nucleus expressing β-galactosidase. The HEK293T indicator target cells were transfected with antisense *env *gene (fusion control), Env71 (non-binding domain), Env144 (RBD) and EnvSU (inhibition control) plasmids. Producer cells were overlaid with HEK293T indicator cells transiently transfected with truncation mutants. Coculture were stained with X-Gal substrate then with May-Grünwald and Giemsa solutions. The determination of the fusion activity of the transfected envelope was performed after 20 h of coculture. The total number of syncytia in one well and the number of nuclei in each syncytia were determined. B) Binding inhibition by an anti-RBD antibody. The Env 144 protein was pre-incubated at 37°C for 1 h with the anti-SU and anti-TM antibodies (dotted line) [5]. Then, XC-hASCT2 cells were incubated at 37°C for 1 h with the protein-antibody complex. The shaded histogram (Env144 incubated with XC-hASCT2) and white histogram (Env144 incubated with parental XC cells) were indicated as controls. The binding was analyzed by flow cytometry with an anti-histidine antibody (anti-RGS(H4)-Mab; Qiagen). C) Receptor binding domain. Variable amino acids (aa) (green dot) among hominoids (upper-case), human (lower case) and neutral insertions preserving the envelope functions (white dots) are indicated above the sequence [23]. Strictly conserved aa (grey boxes) and similar aa (straight line) within the D interference group are underlined. Mutations altering spleen necrosis virus infectivity are indicated as black dots [24]. Blue arrows covering aa 21–69 and 117–144 indicate deletions detrimental to hASCT2 binding. The red arrow corresponds to the SU-EnvW peptide used for rabbit immunization and affinity purification [5].

To formally identify the Env144 protein as a functional RBD product we have evaluated its properties to compete with the wild-type envelope during receptor recognition, using an heterologous cell-cell fusion assay [1,5]. Briefly, TELCeB6 indicator cells expressing the wild-type envelope and characterized by a nucleus expressing β-galactosidase were co-cultured with HEK293T indicator target cells, characterized by uncolored nuclei. The co-culture of both indicator and producer cells leads to the formation of syncytia containing generally one or several blue nucleus and tens white nuclei. The expression in HEK293T of any SU subdomain exhibiting RBD properties would reduce syncytia formation by a receptor interference mechanism. The fusion positive control was characterized by about 730 syncytia per well and an average of 77 ± 42 nuclei per syncytia containing one to ten blue nuclei, as previously observed [1] (Figure 3A). Conversely, expression of EnvSU in HEK293T lead to a decrease in syncytia formation characterized by about 270 syncytia per well and an average of 25 ± 12 nuclei per syncytia containing two to ten blue nuclei, indicating that wild-type envelope could not efficiently interact with hASCT2 receptors on HEK293T cells. Env144 protein expression induces a decrease in fusogenic activity similar to the total SU domain (240 syncytia per well, 26 ± 9 nuclei per syncytia). Conversely, a non-receptor binding protein such as Env71 did not alter fusion efficiency (680 syncytia per well, 66 ± 35 nuclei per syncytia). This demonstrated that the Env144 protein contains all determinants required for hASCT2 receptor binding. In addition, the binding of Env144 and EnvSU to the hASCT1 receptor was found to be similar (data not shown).

The absence of binding in cell surface assays using Env71, Env95 and notably Env117 protein (Figure 2C) suggested the loss of at least one receptor-binding determinant within the 117–144 region. This region contains the epitope of an anti-SU polyclonal antibody (anti-SU raised against residues 112–129 of EnvW) [5]. This antibody was used to detect native syncytin-1 in primary cytotrophoblasts [3] and in b30BeWo carcinoma cells [5]. Hence, the Env144 protein was pre-incubated with the anti-SU antibody. The binding of the protein-antibody complex onto the receptor was significantly altered in the presence of the specific anti-SU antibody but not in the presence of a rabbit anti-TM antibody (Figure 3B). This result indicates that this domain is essential for hASCT2 receptor binding.

In conclusion, we have used HERV-W SU soluble subunits to determine that the 124 N-terminal amino-acids of the HERV-W mature SU protein are sufficient to interact with the hASCT2 and hASCT1 amino-acid transporters (Figure 3C). Although still not elucidated, the envelope-receptor interaction seems to some extent to be tolerant of the RBD amino acid content. Thus, 17 positions out of the 124 residues of the RBD were shown to be variable in ERVWE1 envelopes of hominoids and humans, without altering the hASCT2-dependent fusogenic properties of each envelope variant [4]. In addition, a weak sequence conservation exists between retroviral members of the interference group with the notable exception of three cysteine-containing motifs (PCXC, CYX_5_C and CX_8–9_CW) and a SDGGGX_2_DX_2_R motif. Interestingly, it was shown previously that introduction of 5 residues downstream from serine 51 (see Figure 3C) preserves the fusogenicity of the syncytin-1 protein [23]. This suggests that the first 30 aa of the RBD, that include the conserved PCXC motif, constitute an actual sub-domain. It should be noted that regions of variable sizes are observed between the CYX_5_C and CX_8–9_CW motifs of the retroviruses of the interference group, which suggests a certain flexibility of the interdomain. In addition, the conserved SDGGGX_2_DX_2_R motif seems to be directly involved in receptor interaction, as supported by receptor-binding inhibition with a regio-selective antibody and receptor recognition alteration due to aspartic acid mutated residues in SNV-related retrovirus [24]. Lastly, the carboxy-terminal end of the RBD presents a conserved predicted alpha-helix located immediately upstream of the SDGGGX_2_D sequence. Taken together, these results suggest, for all the retroviruses of the interference group, the existence of 5 structurally or sequence-conserved sub-domains. Crystallography studies will be required to confirm that these sub-domains interact. Finally, as the syncytin-1 belongs to the HERV-W family, many natural HERV-W truncated envelope ORFs probably contain the RBD. When reactivated, these partial envelopes could interfere with the hASCT receptors, which suggests putative physiopathological functions for such truncated envelopes.

## Abbreviations

HTLV 1 and 2: Human T-cell leukemia virus 1–2

HERV-W: human endogenous retrovirus W

RDR: type D mammalian retrovirus receptor

SU: surface subunit

TM: transmembrane subunit

Env: envelope glycoprotein

His: histidine

EGF: Epidermal growth factor

Mab: monoclonal antibody

## Competing interests

The author(s) declare that they have no competing interests.

## Authors' contributions

V.C and O.G designed this study and V.C edited the manuscript. They also participated in the construction of mutants, cell culture, transfection, and FACS analyses. F.M conceived the study, participated in its design and coordination and helped draft the manuscript.
